# Transparent, Open, and Reproducible Prevention Science

**DOI:** 10.1007/s11121-022-01336-w

**Published:** 2022-02-17

**Authors:** Sean Grant, Kathleen E. Wendt, Bonnie J. Leadbeater, Lauren H. Supplee, Evan Mayo-Wilson, Frances Gardner, Catherine P. Bradshaw

**Affiliations:** 1 Department of Social & Behavioral Sciences, Fairbanks School of Public Health, Indiana University Richard M, 1050 Wishard Blvd, Indianapolis, IN 46202 USA; 2 Department of Human Development and Family Studies, Colorado State University, Fort Collins, CO USA; 3 Department of Psychology, University of Victoria, Victoria, BC Canada; 4 Child Trends, Bethesda, MD USA; 5 Department of Epidemiology and Biostatistics, Indiana University School of Public Health-Bloomington, Bloomington, IN USA; 6 Department of Social Policy and Intervention, University of Oxford, Oxford, UK; 7 School of Education & Human Development, University of Virginia, Charlottesville, VA USA

**Keywords:** Open science, Prevention, Replication, Reproducibility, Research transparency

## Abstract

The field of prevention science aims to understand societal problems, identify effective interventions, and translate scientific evidence into policy and practice. There is growing interest among prevention scientists in the potential for transparency, openness, and reproducibility to facilitate this mission by providing opportunities to align scientific practice with scientific ideals, accelerate scientific discovery, and broaden access to scientific knowledge. The overarching goal of this manuscript is to serve as a primer introducing and providing an overview of open science for prevention researchers. In this paper, we discuss factors motivating interest in transparency and reproducibility, research practices associated with open science, and stakeholders engaged in and impacted by open science reform efforts. In addition, we discuss how and why different types of prevention research could incorporate open science practices, as well as ways that prevention science tools and methods could be leveraged to advance the wider open science movement. To promote further discussion, we conclude with potential reservations and challenges for the field of prevention science to address as it transitions to greater transparency, openness, and reproducibility. Throughout, we identify activities that aim to strengthen the reliability and efficiency of prevention science, facilitate access to its products and outputs, and promote collaborative and inclusive participation in research activities. By embracing principles of transparency, openness, and reproducibility, prevention science can better achieve its mission to advance evidence-based solutions to promote individual and collective well-being.

## Transparent, Open, and Reproducible Prevention Science

The field of prevention science aims to generate reliable evidence concerning the etiology of and responses to educational, health, and social issues—and to translate that evidence into policies and practices to promote individual and collective well-being (Botvin, 2000). The potential influence of prevention science on significant societal problems and inequities demands a high level of scientific rigor and research integrity (Catalano et al., 2012). Consequently, prevention scientists have worked to establish methodological and ethical standards that yield valid and actionable evidence (Crowley et al., 2018; Flay et al., 2005; Gottfredson et al., 2015; Leadbeater et al., 2018; Spoth et al., 2013). As it has done previously, prevention science continues to revisit its standards and norms in response to new opportunities and concerns, starting with constructive discussion and debate.

There is growing interest in prevention science and related disciplines in the transparency, openness, and reproducibility of scientific research (see Table 1). This movement—most commonly referred to as “open science”—aims to make the scientific process public and auditable, and to ensure the free availability and usability of scientific knowledge (Bezjak et al., 2018). Examples of open science practices include registering studies, protocols, and analysis plans; sharing data, analytic methods, and other research materials; reporting and disclosing all study methods and findings; and disseminating research outputs via open access outlets (for a more complete review, see (Miguel et al., 2014; Munafò et al., 2017; Nosek et al., 2015). As awareness and support of these practices has increased in recent years (Christensen et al., 2020), conversations in most disciplines have evolved from “whether” open science should be the norm to “how” to implement transparent, open, and reproducible research practices (National Academies of Sciences, 2018, 2019). Notably, as fields transition to open science, researchers often raise pragmatic concerns about the potential for additional burdensome bureaucracy and regulation, stifled creativity and discovery, and inappropriate application to studies not based on hypothetico-deductive models (Academy of Medical Sciences, 2015).

**Table 1:** Glossary

**Term**	**Definition**	**Reference**
Analysis Plan	Technical, detailed elaboration of the procedures for executing the analysis described in the protocol	Gamble et al. (2017)
Availability Standards	Guidelines for making data, analytic code, and research materials findable, accessible, interoperable, and reusable	Nosek et al. (2015)
Dynamic Documents	Documents combining code, rendered output, and prose that can be continually edited and updated	Xie et al. (2020)
Inferential Reproducibility	Making knowledge claims of similar strength from a study replication or reanalysis	Goodman et al. (2016)
Methods Reproducibility	Ability to implement study procedures as exactly as possible, with the same data and tools, to obtain the same results	Goodman et al. (2016)
Open Access	Free, immediate, online availability of research articles, with copyright that allows sharing and adaptation	Tennant et al. (2016)
Open Notebook	Practice of making the primary record of a research project publicly available (e.g., online as it is recorded)	Schapira and Harding (2020)
Open Source	Software source code released with a copyright that allows use, adaptation, and distribution for any purpose	Peng (2011)
Preprints	Version of a scientific manuscript posted on a public server prior to formal peer review	Sarabipour et al. (2019)
Protocol	Document with comprehensive details on study background, rationale, objectives, design, and methods	Chan et al. (2013)
Registered Reports	Publishing format in which protocols undergo peer review, followed by in-principle acceptance of the results paper	Chambers (2013)
Reporting Standards	Minimum set of study information needed for an accurate, complete, and transparent account of what was done and found	Simera et al. (2010a, 2010b)
Research Lifecycle	Stages of a research study, such as prioritization, design, conduct, reporting, and overall management of a research study	National Academies of Sciences (2018)
Results Reproducibility	Production of corroborating results in a new study, having followed the same methods as the original study	Goodman et al. (2016)
Scientific Ecosystem	Interacting community of scientific stakeholders and their environments	Moher et al. (2016)
Study Registration	Process of entering a minimum dataset about an empirical study in an independently controlled registry that is accessible to the public	De Angelis et al. (2005)
Version Control	System that records changes to files over time in a way the facilitates later recall of specific file versions	Gentzkow and Shapiro (2014)
Workflow	Management and organization of folders, files, metadata, analytic code, and other study data documentation	Project TIER (2016)

The Society for Prevention Research (SPR) has identified the relevance of specific open science practices in prior work, such as task forces on standards of evidence for prevention interventions (Gottfredson et al., 2015) and ethical issues encountered by prevention scientists (Leadbeater et al., 2018). More recently, a featured roundtable session at the 2019 SPR Annual Meeting explicitly focused on open science within prevention science (Bradshaw et al., 2019). To promote further discussion on this critical issue, the panel participants and session attendees recommended that *Prevention Science* publish a special issue on transparency, openness, and reproducibility—which three session participants and co-authors of this manuscript (SG, FG, and CPB) subsequently pursued. This paper serves as a primer introducing and reviewing key concepts for this special issue, with subsequent papers providing deeper analysis on the implications of specific concepts to prevention science. In this paper, we review the opportunities and concerns motivating the wider open science movement. We also consider core practices, resources, and stakeholders involved in advancing an open science reform effort, with attention to the intersection of open science practices and prevention science methods. We conclude with some challenges to consider in future discussions about the transition to a transparent, open, and reproducible prevention science.

## Factors Motivating the Open Science Movement

Proponents of open science advocate for transparency, openness, and reproducibility as mechanisms to align scientific practice with scientific ideals, accelerate scientific discovery, and broaden access to scientific knowledge (National Academies of Sciences, 2018, 2019). Depending on the nature and importance of a study, these principles are operationalized as one or more relevant open science practices (Mayo-Wilson & Dickersin, 2018). In this section, we summarize these factors motivating our call for concerted efforts to align prevention science with the open science movement.

### Aligning Scientific Practice with Scientific Ideals

Transparency, openness, and reproducibility are inherent in fundamental scientific ideals, such as communality, universalism, disinterestedness, and organized skepticism (Merton, 1973). For example, open science practices better enable researchers to verify the work of others. Verifiability relates to the ideal of science as “self-correcting,” which means the scientific community governs itself in order to calibrate evidentiary claims and limit unavoidable errors, thereby safeguarding credibility and instilling trust in the scientific literature (Vazire, 2018). Because verifiability requires researchers to provide empirical support for scientific claims, practices like data sharing enable the verifiability of empirical evidence. Toward that end, open science bolsters research integrity by facilitating verifiability. As researchers increasingly espouse these ideals, making open science the norm would better align actual scientific practice with the ideals to which scientists subscribe (Anderson et al., 2016; Anderson et al., 2010).

### Accelerating Scientific Discovery and Progress

Open science also can accelerate the progress of science as a cumulative enterprise. Transparency and reproducibility facilitate reuse and building on the work of others, leading to greater returns on research investments (Academy of Medical Sciences, 2015). For example, sharing data, code, and materials allows a greater proportion of products from previous research to influence new studies (Goodman et al., 2016). These practices can speed the process of new discoveries and expedite error detection, thereby redirecting unproductive lines of research more quickly (Vazire, 2018). A new research team can better check the internal consistency of another team’s results, reanalyze data using the original analytical strategy, and examine robustness to alternative analytical choices, prior to conducting a new study (Nuijten, 2022). In addition, openness enables collaborations not possible through siloed research, such as crowdsourced initiatives that build large datasets to create opportunities for a greater number of rich data analyses (Moshontz et al., 2018). Data sharing also yields greater power to investigate new or more complex questions (e.g., intervention effects on rare outcomes, subgroup effects, or moderated mediation) that require larger sample sizes than are typically found in one study (Leijten et al., 2020). Adopting protocols, software, and analytic strategies from previous studies can increase standardization, facilitating more efficient discoveries and research syntheses that summarize the cumulative evidence within a line of scientific inquiry (Goodman et al., 2016).

### Broadening Access to Scientific Knowledge

The open science movement also focuses on making research products and outputs more usable and freely available to everyone, broadening access to scientific knowledge and resources. For example, disparities in financial, human, and physical resources across research institutions can be mitigated by the free availability and reuse of protocols, data, code, software, and materials from previous research (Gennetian et al., 2020). In addition, open access articles can be read online or downloaded freely by stakeholders not affiliated with research institutions that have journal subscriptions, such as non-governmental organizations, policymakers, and engaged citizens. Through this focus on free availability of research findings and products, open science can accelerate the flow of scientific evidence to the public.

## Need for an Open Research Lifecycle

Researchers make numerous decisions across all stages of research, or the research lifecycle, including question formulation, study design, data collection and analysis, and reporting and dissemination (National Academies of Sciences, 2018). Without transparency, researchers have undisclosed flexibility in making these decisions (sometimes called “researcher degrees of freedom”), which enable specific concerns motivating the open science movement (Wicherts et al., 2016). For example, a “closed” research lifecycle hinders the ability to reproduce previous research (Goodman et al., 2016), facilitates selective non-reporting of studies and results (i.e., publication bias and outcome reporting bias) and other detrimental research practices (Dwan et al., 2013), prevents detection of unintentional errors and intentional misconduct (Fanelli, 2009), and exacerbates perverse incentives for career scientists (Smaldino & McElreath, 2016). In this section, we consider some of the concerns and challenges for the field of prevention science that can be addressed by adopting open science.

### The “Reproducibility Crisis”

Over the last decade, scientists and other stakeholders have contended that the behavioral, social, and health sciences are experiencing a “reproducibility crisis” (Fidler & Wilcox, 2018). Numerous large-scale collaborative efforts have found low reproducibility rates in psychology (Klein et al., 2014; Open Science, 2015), economics (Camerer et al., 2016; Chang & Li, 2015), the social sciences (Camerer et al., 2018), and medicine (Nosek & Errington, 2017). While irreproducibility can occur for substantive reasons, scientific stakeholders are concerned that the number of key research findings that cannot be reproduced is higher than expected or desired, particularly in high-profile scientific journals (Shrout & Rodgers, 2018). Viewing ability to reproduce findings as one indicator (of many) for the truth of a scientific claim (Goodman et al., 2016), these results are commonly taken as evidence that a greater proportion of published research findings are likely false than has previously been believed (Baker, 2016; Gall et al., 2017). A high proportion of false research findings can hinder scientific progress, delay translation of research into policy and practice applications, lead to waste of resources, and threaten the reputation of and public trust in science (Academy of Medical Sciences, 2015).

Goodman et al. (2016) offer a three-term taxonomy that may be helpful to facilitate shared understanding of and clear communication about reproducibility within prevention science. First, “methods reproducibility” refers to the ability to implement the same methodological and computational procedures with the same data to obtain the same results as a previous study. It facilitates trust that data and analyses are as represented, requiring provision of enough detail about original study methods and data for another to repeat the same procedures. “Results reproducibility” refers to the ability to implement the same methodological procedures with a new, independent dataset to produce results corroborating a previous study. Using this terminology, a replication study generally refers to a study designed to examine or test the results reproducibility of a previous study, with the potential to provide new evidence for a scientific claim (Academy of Medical Sciences, 2015). Finally, “inferential reproducibility” refers to the ability to draw conclusions that are qualitatively similar to a previous study, either from an independent replication or reanalysis of the original study data. All three types of reproducibility are relevant to the field of prevention science and germane to the open science movement, each with important considerations across stages of the research lifecycle.

### Detrimental Research Practices

While there are various determinants of reporting findings that are false and irreproducible, common “detrimental research practices” may be important contributors (Munafò et al., 2017). Some researchers intentionally engage in these practices with full knowledge of their negative consequences; however, most researchers likely do so unknowingly or under the belief that these practices are acceptable and compatible with research integrity (John et al., 2012). Regardless of intention or understanding, these practices have detrimental effects on research integrity by inflating the false positive error rate in the research literature (National Academies of Sciences, 2017; Simmons et al., 2011). Unfortunately, evidence suggests that many of these practices are not only common, but may be increasing over time (Chavalarias et al., 2016; Fanelli, 2012; Masicampo & Lalande, 2012; Pocock et al., 2004).

Chief among these detrimental research practices is selective non-reporting of studies and results, which occurs when the nature of study findings (rather than methodological rigor) influences the decision to submit, disseminate, or publish them (Chalmers, 1990; Chan, 2008). There is ample and long-standing evidence across disciplines that “statistically significant” or “positive” results are more likely to be published than results that are “not statistically significant,” “negative,” “null,” “inconclusive,” or otherwise countervailing (Axford et al., 2020; Dwan et al., 2013; Fanelli, 2010a, 2010b; Franco et al., 2014; Hartgerink et al., 2016; Sterling, 1959). Researchers may selectively refrain from writing-up and submitting entire studies for publication based on the nature or direction of results (Rosenthal, 1979), leading to a biased subsample of studies being published in the literature on a research topic. For example, selective non-reporting of entire studies (“publication bias”) has been documented in psychology and education using evidence from institutional review boards and doctoral dissertations that shows studies with statistically nonsignificant results are less likely to be published (Cooper et al., 1997; Pigott et al., 2013). In clinical psychology and medicine, interventional trials with statistically nonsignificant results are less likely to be published than clinical trials with statistically significant results (Cuijpers et al., 2010; Cuijpers et al., 2010; Dwan et al., 2013; Niemeyer et al., 2012, 2013; Song et al., 2010). An evaluation of trials funded by the National Institute of Mental Health found that studies with small effects were less likely to be published than studies with large effects, inflating the apparent effectiveness of psychotherapies (Driessen et al., 2015).

There is also selective non-reporting of study results (“reporting bias” or “selective outcome reporting”), which occurs when researchers choose a subset of outcomes to report in manuscripts (Axford et al., 2020; Chan et al., 2004). Selective non-reporting of results may be difficult to detect because it tends to be apparent only when study protocols and statistical analysis plans are registered prospectively, and when reviewers or readers check published results against registered outcomes and analyses. Nonetheless, there is also empirical evidence that statistically nonsignificant results are more likely to be omitted selectively from manuscripts (Chan & Altman, 2005; Staines & Cleland, 2007). In contrast, selective non-reporting of whole studies may be more apparent, especially in the case of large prevention trials. Scientific claims based on these bodies of evidence are undermined as a result of authors, journal editors, and peer reviewers using the statistical significance, magnitude, or direction of results to make publication decisions (Dickersin, 1992; Emerson et al., 2010; Olson et al., 2002).

Selective non-reporting is related to other detrimental research practices that virtually guarantee (spuriously) finding statistically significant or interesting results (Goodman et al., 2016). For example, “p-hacking” refers to repeatedly searching a dataset or trying multiple alternative analyses until a statistically significant or desired finding is obtained—and then failing to fully report how this result was obtained (Simonsohn et al., 2014). “Hypothesizing After Results are Known,” or “HARKing,” involves reporting a hypothesis formed after seeing study results as if it were an a priori hypothesis formed before collecting or analyzing study data (Kerr, 1998). Undisclosed flexibility in the research lifecycle also can hinder the ability of peer-reviewers and readers to detect research practices that result in overfitted statistical models, i.e., overly optimistic “findings” from the statistical model of a dataset that do not occur in the target population due to idiosyncrasies of the sample at hand (Babyak, 2004). Spurious findings from overfitted statistical models (such as linear regressions, logistic regressions, structural equation models, and other models common in prevention sciences) are highly likely to fail to replicate in future samples, threatening the credibility of scientific claims supported by these findings. In addition, overinterpretation and misuse of inferential statistics can occur in cases of low statistical power (Button et al., 2013; Szucs & Ioannidis, 2017), lenient and arbitrary thresholds for statistical significance (Benjamin et al., 2018; Lakens et al., 2018), errors in reporting p-values (Nuijten et al., 2016), and inappropriate application of null hypothesis significance testing (Goodman et al., 2016).

While more rare than the aforementioned detrimental research practices, high-profile cases of intentional research misconduct have also generated recent interest in reproducibility (Stroebe et al., 2012). These intentional practices include fabrication, falsification, and plagiarism (National Academy of Sciences, National Academy of Engineering, & Institute of Medicine, 1992). Fabrication involves making up data or results, while falsification involves misrepresenting research through the manipulation of materials or data. In contrast, plagiarism is appropriating another person’s work without due credit when proposing, performing, or reporting research. As human beings, researchers also make honest technical or human errors—such as model misspecification or data entry errors in a spreadsheet (Academy of Medical Sciences, 2015). Whether intentional or not, closed research lifecycles hinder the ability to detect these issues that negatively impact the validity of reported research findings.

## An Overview of Core Open Science Practices

**Fig. 1:**
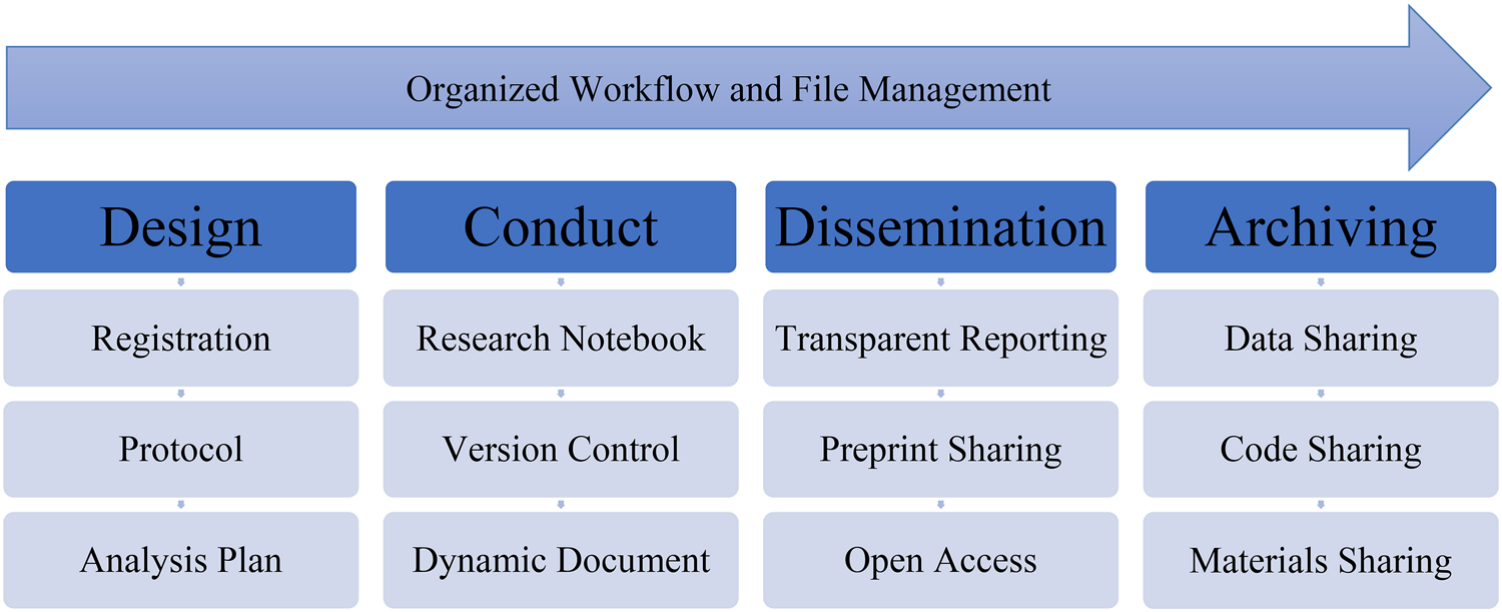
Roadmap for a Transparent, Open, and Reproducible Research Lifecycle**. **Note: Figure adapted from the roadmap co-developed by SG for the Berkeley Initiative for Transparency in the Social Sciences Research Transparency and Reproducibility Training (RT2) workshops: https://www.bitss.org/resource-library/

In response to what has been called the *reproducibility crisis*, the open science movement represents part of what is being called a *credibility revolution* (Spellman, 2015), by promoting standards and norms that increase the reliability of scientific research (Goodman et al., 2016; Vazire, 2018). One of many scientific reform efforts (Munafò, 2019), open science aims to promote a shift from traditionally closed to more open research lifecycles (Miguel et al., 2014; Nosek et al., 2015). To achieve this shift, open science proponents commonly advocate for a core set of practices (see Fig. 1).

**Fig. 2:**
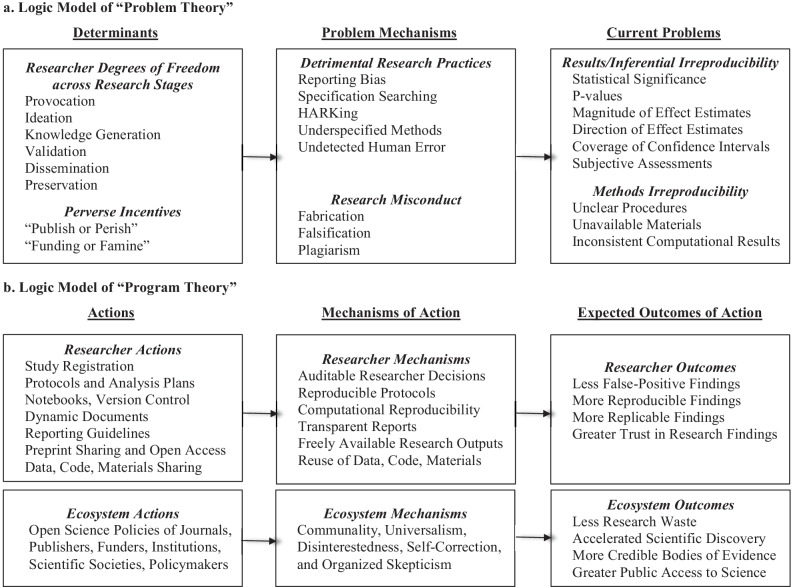
Logic Models of Open Science "Problem Theory" and "Program Theory"

It is important to note, however, that this set of core open science practices has largely arisen from idealized versions of studies using the hypothetico-deductive scientific method, such as randomized trials and experiments testing confirmatory analyses via null hypothesis significance testing in a frequentist statistical framework (Munafò et al., 2017). While principles of transparency and openness are relevant to all empirical research in prevention science, it may be premature or undesirable to require each open science practice for every type of empirical study a prevention scientist might conduct. Rather, a goal of this paper is to provide readers (particularly those new to transparency and reproducibility) with an overview of prominent open science practices. In turn, this article (and the Special Issue of *Prevention Science* that it anchors) can serve as a foundation for further discussion about the various elements of open science that can be applied in prevention science. In the sections that follow, we describe open science practices in greater detail, along with a description of the stakeholders and contexts of research that contribute to a need for greater openness and transparency. We then connect these activities and concepts to prevention science research practices.

### Study Registration

Study registration is a time-stamped entry of a minimum set of information in a publicly accessible, independently controlled registry (De Angelis et al., 2005). Researchers may register their studies before collecting new data or accessing existing datasets (De Angelis et al., 2004). Study registration can address publication bias by documenting that particular studies exist, and they can serve as “identification numbers” that link various products and outputs of a study, such as protocols, data, code, research materials, and manuscripts (Altman et al., 2014). In this way, a study registration acts as a “one-stop shop” for other researchers and interested stakeholders to discover and gather information on planned, current, and completed research on a topic, even if that research is unpublished (Harrison & Mayo-Wilson, 2014).

### Protocols and Analysis Plans

A protocol is a document with details on study background, rationale, objectives, design, and methods (Chan et al., 2013). An analysis plan provides a technical and detailed elaboration of the procedures for executing the analysis described in the protocol (Gamble et al., 2017). Researchers can register, publish, or share these documents in advance of data collection and analysis in order to pre-specify the rationale, proposed methods, analysis plan, ethical considerations, and management of a research study (Nosek et al., 2012). Protocol and analysis plan registration does not prevent exploratory analyses (Wagenmakers et al., 2012). Rather, prospective registration limits opportunities for detrimental research practices (e.g., outcome switching, selective outcome reporting, and HARKing) by facilitating external identification of planned versus actual study procedures and analyses (Goodman et al., 2016). Protocols and analysis plans also encourage research teams to more carefully plan in advance (Nosek et al., 2018). They also help research teams to conduct the study; human research protection programs to assess the risks and benefits of proposed study procedures; and research consumers to monitor and evaluate changes throughout the research lifecycle (Tetzlaff et al., 2012).

### Organized Workflows

Study workflow involves folders, files, metadata, code for analyses, and other data documentation (Project TIER, 2016). Study workflows can be organized coherently and document file management procedures clearly (Long, 2008). A reproducible workflow includes (a) clear computing and communication, (b) version control that tracks changes in real time across collaborators and versions (ideally with a cloud-based mirror), (c) tracking the chronology and origin of research objects (e.g., data, source code), (d) maximum programmatic automation and minimal manual file edits, and (e) containment of a computational environment to share with others who would like to repeat the workflow (Martinez et al., 2020). Ideally, researchers maintain a dynamic, digital notebook that records decisions made throughout the research lifecycle, and make this notebook publicly available after study completion (Schapira & Harding, 2020). In addition, a well-commented markdown file can capture which and how many analyses were performed and ultimately reported in published manuscripts (Goodman et al., 2016).

### Transparent Reporting

Incomplete reporting leads to the omission of information essential to appraise study quality, reproduce findings, and synthesize a body of evidence (Grant et al., 2013). Reporting guidelines use explicit methodology to provide standards on the minimum set of study information to include in a manuscript for an accurate, complete, and transparent account of what was done and found in a study (Moher et al., 2010). These reporting standards are organized in a checklist according to the introduction, methods, results, and discussion sections of an article, as well as a diagram for capturing the flow of participants through study stages (Simera et al., 2010a, 2010b). The EQUATOR (Enhancing the QUAlity and Transparency Of health Research) Network is an international initiative that provides a catalog of reporting guidelines for various study designs, as part of its mission to improve the quality of scientific publications through transparent and accurate reporting (Simera et al., 2010a, 2010b).

### Data, Code, and Materials Sharing

Sharing analytical datasets with relevant metadata, code, and related research materials facilitates reproducibility. To safeguard quality, researchers can carefully plan and describe management procedures at the beginning of a study, make those procedures accessible to the research team during the study, and communicate these procedures to external stakeholders after the study (Nosek et al., 2015). Data relevant for sharing can range from initial raw data to the final processed dataset—given the many judgments and choices are made by study teams along the path of cleaning, transformation, and preparation for analysis (Goodman et al., 2016). Availability standards offer guidelines for managing data, code, and research materials, and for making them findable, accessible, interoperable, and reusable (Wilkinson et al., 2016). Sharing allows reported findings to be verified directly through reproducibility and sensitivity checks, as well as enable other investigators to pursue new questions in secondary data analyses (Gilmore et al., 2018). Barring legal, ethical, and proprietary constraints, researchers can share their data, code, and materials in permanent repositories dedicated to archiving, such as GitHub, Dataverse, Dryad, Vivli, and the Interuniversity Consortium for Political and Social Research (Christensen et al., 2019).

## Stakeholders in the Scientific Ecosystem

Researcher behaviors leading to reproducibility problems are influenced by aspects of the current context, culture, and incentive structure of scientific careers (Fidler & Wilcox, 2018). Incentives for academic researchers have become increasingly perverse over the last several decades because of the hyper-competitive research environment and its focus on productivity, novelty, and innovation (Edwards & Roy, 2017). Competition for publications, research funding, media coverage, and permanent employment can incentivize detrimental research practices (Smaldino & McElreath, 2016), creating a tension between career advancement and the credibility of the published scientific literature (Nosek et al., 2012). The resulting “publish or perish” and “funding or famine” culture—in combination with “closed” research lifecycles—is a key determinant of the proportion of false positives, irreproducible results, and detrimental research practices in the scientific literature (Moher et al., 2018). Given that these systemic factors facilitate detrimental research practices, cultural changes are needed to improve the reproducibility of the scientific literature. Consequently, open science reform efforts target not only researcher behaviors, but also focus on changing the scientific ecosystem, i.e., the way that scientific stakeholders, the wider research environment, and the resultant incentives interact as a system (Moher et al., 2016). Several specific stakeholders in the scientific ecosystem are commonly targeted to encourage researcher adoption of open science practices (see Table 2). Engaging multiple stakeholders in promoting transparency, openness, and reproducibility is particularly important for decentralized fields like prevention science that have less regulations compared to biomedicine (Dal-Ré et al., 2015; Faggiano et al., 2014).

**Table 2:** Proposed Stakeholder Actions for Supporting Open Science

**Stakeholder**	**Proposed Action**	**Reference**
Researchers	Adopt transparent, open, and reproducible research practices in empirical prevention science	Christensen et al. (2019)
Universities and Research Institutions	Explicitly support, recognize, and reward the use of transparent, open, and reproducible practices by faculty and researchers	Moher et al. (2019)
Students, Postdocs and Early Career	Enroll in courses and training in open science practices, build coalitions for peer support, and incorporate open science into daily lab work, dissertations, and research	Morling and Calin-Jageman (2020)
Journals and Publishers	Implement policies and procedures that promote publishing articles of transparent, open, and reproducible research	Nosek et al. (2015)
Funders	Promote or mandate adherence to open science practices in grant applications and funded projects	National Academies of Sciences (2018)
Scientific Societies	Advance the use of transparent, open, and reproducible research practices in guidance, conference proceedings, and trainings	McVay and Conroy (2019)
Practitioners	Advocate for transparent, open, and reproducible research practice in empirical scientific studies of and standards for establishing evidence-based interventions	Mayo-Wilson et al. (2020)
Policymakers	Incorporate transparent, open, and reproducible research practices into policy analysis and standards of evidence	Hoces de la Guardia et al. (2021)
Media	Incorporate considerations and standards related to transparency, openness, and reproducibility when reporting about science to the public	Academy of Medical Sciences (2015)
The Public	Participate and collaborate in scientific research to increase scientific knowledge and address problems of concern in their local communities	Chari et al. (2019)

### Journals and Publishers

As dissemination of research through journal articles influences career opportunities, peer review and publication models are important for the open science movement. Journals have been criticized for traditional publication models that focus on novelty rather than reproducibility, results rather than methods, and narrative rather than data and analysis (Fidler & Wilcox, 2018; Neuliep & Crandall, 1990; Schmidt, 2009). In addition, publishers are a stakeholder group distinct from journals that create their own policies and procedures that shape research practice and career incentives for scientists (Nosek et al., 2015). For example, publishers influence (and in many cases prescribe) the standards, language, and format of the instructions for authors pages of their journal websites. They also set the fields and functionalities of article submission systems, review systems, and the templates for journal articles and their landing webpages (Mayo-Wilson et al., 2021). Moreover, active debate abounds regarding for-profit versus non-profit publishers, open access fees, and relevant consequences on the representativeness and welfare of the research community (McNutt, 2019). To address these observations and concerns, publishers can enable journal editors to adopt policies and procedures that promote transparency, openness, and reproducibility of the science that they publish (Azar et al., 2015; Cybulski et al., 2016; Grant et al., 2013; Knüppel et al., 2013; Milette et al., 2011; Riehm et al., 2015; Scott et al., 2015).

The Transparency and Openness Promotion (TOP) Guidelines comprise eight modular standards that journals can incorporate into their policies for manuscript submission and publication (Nosek et al., 2015). In tandem with the TOP Guidelines, some journals award digital “open science badges” to manuscripts that involve these practices, such as data sharing, materials sharing, or study registration (Kidwell et al., 2016). Furthermore, journals can offer Registered Reports: a two-step submission process where the protocol is reviewed prior to conducting the research, with in-principle acceptance of the subsequent results papers should second-stage review confirm that any deviations from the approved protocol are justifiable (Chambers, 2013). In addition to addressing publication biases, this model also allows feedback on the protocol to improve the actual conduct of the study, as changes to design and conduct can still be incorporated and lead to a more constructive peer review process (Chambers, 2019). To complement Registered Reports, journals can offer “Exploratory Reports” for empirical submissions that address relatively open research questions using abductive and inductive approaches without strong a priori predictions (McIntosh, 2017). Offering Exploratory Report and Registered Report formats would respect both the exploratory and confirmatory phases of discovery vital to prevention science. Lastly, to increase the transparency of the review process itself, journals and publishers are increasingly trialing “open peer review models,” including making reviewer and author identities known, publishing review reports alongside articles, and crowdsourcing participation in the peer review process (Ross-Hellauer, 2017).

To facilitate accessibility and more efficient discovery of their completed work, journals can have options for authors to publish open access manuscripts, as well as post working versions or *preprints* of submitted papers (Tennant et al., 2016). Open access publication involves free and immediate online availability of research articles, with copyright that allows sharing and adaptation (Tennant et al., 2016). Preprints are publicly available scientific manuscripts posted on dedicated servers prior to journal-managed peer review (Sarabipour et al., 2019). While benefits of preprints include more rapid dissemination of and feedback on academic work, concerns include sharing and subsequent media coverage of substandard work with significant implications (Kaiser, 2017). Journals and publishers vary in their policies on open access publishing and pre-print sharing, with evidence to suggest a growing number of journals with options for both practices (da Silva & Dobránszki, 2019; Laakso, 2014).

### Funders

As grants and contracts also influence career opportunities, funders can implement policies and procedures to promote the transparency, openness, and reproducibility of the research that they sponsor. To ensure maximal return on their investments, funders could require that researchers be transparent about their procedures and share all products of their funded scientific research (Gennetian et al., 2020). For example, the San Francisco Declaration on Research Assessment (2018) recommends considering the value and impact of research outputs beyond publications—such as datasets, software, computational environment, and code—when evaluating the scientific productivity of grant applicants. The National Institutes of Health has policies that set explicit expectations on sharing data, open access publication, and registering and sharing results of clinical trials. Funders also can have specific “requests for proposals” related to transparency, openness, and reproducibility. For instance, the Institute of Educational Sciences (2021) has a dedicated request for applications on systematic replication studies that vary one or more aspects of a previous study to better understand which interventions improve education outcomes, for whom, and under what conditions. Funders also can dedicate resources to infrastructure, training, and staff required for open science practices, such as the National Institute of General Medical Sciences (2018) clearinghouse of training modules to enhance reproducibility. Such dedicated resources are essential, given robust evidence that actual rates and quality of data sharing by principal investigators is suboptimal, even when support for and willingness to share data are high (Ohmann et al., 2021).

### Universities and Research Institutions

Universities and research institutions also influence researcher behaviors. Given their role in enabling perverse “publish or perish” and “funding or famine” incentives (Bouter, 2020), open science proponents are increasingly calling on universities and research institutions to empower researchers to stop using detrimental research practices (Woolston, 2021) and to normalize committees to reward transparent, reproducible research practices for career advancement (Moher et al., 2020). Hiring, promotion, and tenure assessments of faculty at universities could reward transparently publishing all research results and openly sharing data, code, protocols, and other research materials (Moher et al., 2018). Universities also can provide training on open science practices through formal coursework on transparency, openness, and reproducibility for graduate students and postdoctoral fellows (Krishna & Peter, 2018), as well as support through fostering Open Science Communities at their institutions (Armeni et al., 2021). Given the costs involved in learning new knowledge and skills, universities and research institutions also can seek mechanisms to provide their students, faculty, and researchers with protected funding and time to develop proficiency in open science practices, such as resources to support data archiving (Gilmore et al., 2020). Universities also could consider leveraging existing research administration and quality assurance offices—such as clinical trials offices (Mayo-Wilson et al., 2018) and human subjects research protection programs (Grant & Bouskill, 2019)—to help facilitate the transparency, openness, and reproducibility of ongoing research. Lastly, universities and research institutions can adopt policies signaling support for open science. For example, several research institutions—including Child Trends (https://www.childtrends.org/policies-on-integrity-independence-and-transparency), the International Initiative for Impact Evaluation (https://www.3ieimpact.org/our-work/research-transparency), and MDRC (https://www.mdrc.org/publication/research-transparency-and-replication-mdrc)—have created research transparency policies that support practices such as study registration, data archiving, and open access publication. The National Academies of Sciences (2021) recently developed an extensive toolkit of resources that universities and research institutions can use to foster open science.

### Policymakers and Practitioners

Policymakers and practitioners would benefit from the more efficient scientific discoveries and accessible evidence afforded by transparency, openness, and reproducibility. For example, incorporating open science practices into the standards used by clearinghouses to designate interventions as “evidence-based” could influence researchers to use these practices in program evaluations, as well as lead to an even more reliable evidence-base for decision-making (Buckley et al., 2021; Mayo-Wilson et al., 2020). Federal agencies that oversee policy and program evaluation efforts have demonstrated a growing interest in open science methods as critical to fulfilling obligations to be a steward of and efficiently use taxpayer dollars (Holzwart & Wagner, 2020). The Administration for Children and Families (2014) has created an evaluation policy that includes a commitment to transparency and openness via publishing study plans in advance, comprehensively presenting all results, and making timely information about planned, ongoing, and completed evaluations easily accessible. The Office of Evaluation (2020) likewise publishes analysis plans prospectively, and it provides resources on pre-registration of and handling null results from program evaluations. Furthermore, the U.S. Department of Agriculture requires contractors to adhere to specific data management processes and then reviews these processes all materials to ensure compliance (Burdg, 2019). In addition, the Foundations for Evidence-Based Policymaking Act of 2018 (P.L. 115–435) includes requirements related to transparency and openness of federal research and evaluation—including public-facing annual evaluation plans, open data, and data inventories—as part of enhancing federal government capacity for evidence building. These practices will lead to more credible and useful evidence on policies and programs that directly impact prevention efforts.

### Media and the Public

Open science also facilitates the inclusion of media and the public in the scientific enterprise. Issues of reproducibility in science recently have garnered attention in popular media (Harris, 2017; Yong, 2018). Engaging the media as part of open science efforts can facilitate better communication about the scientific process in the popular press (Academy of Medical Sciences, 2015; Sumner et al., 2014). In addition, open science offers unique opportunities for public engagement in research. The new paradigm of “citizen science” allows members of the general public to collect scientific data for freely available datasets that provide actionable information for their local community (Chari et al., 2019). These practices provide promising mechanisms for improving public discourse on and trust in science.

## Applying Open Science to Prevention Science

Each field—including prevention science—has its own standards, approaches, methods, and culture that need to be considered in reform efforts (Academy of Medical Sciences, 2015). The problems addressed by and implementation of specific open science practices vary in relevance across different phases of prevention research. In this section, we consider different types of prevention science research and ways in which they can adopt elements of open science. While not intended to serve as formal standards for the field, they may serve as a foundation for discussion about and the creation of such standards and recommendations for different types of prevention science research by an established task force or working group (Hesse et al., 2021).

### Epidemiology and Etiology

A core aspect of prevention science is the investigation of the distribution and causes of physical, mental, and social health problems among populations. Epidemiological research within prevention science may be at greater risk of multiple hypothesis testing, and the selective non-reporting of studies and results, because of increased capacity to fit increasingly complex models (Goodman et al., 2016). Non-reporting of results from epidemiological research wastes resources, and can increase the chances that the wrong risk and protective factors are pursued in future intervention research (Chan et al., 2014; Glasziou et al., 2014). Project management systems, such as the Open Science Framework, offer prevention scientists conducting epidemiological research with free, open, and online platforms to collaboratively organize workflows, manage files, and share notebooks (Foster & Deardorff, 2017). In addition, using reporting guidelines such as the Strengthening the Reporting of Observational Studies in Epidemiology (STROBE) Statement (von Elm et al., 2014) can lead to more transparently disseminated observational studies, of which most epidemiological research consists.

### Development and Testing of Interventions

The development and testing of interventions are fundamental to prevention science. From an open science perspective, study registration and transparent reporting are essential practices for these stages of research. Trial registration involves recording important information about trial design, particularly complete and transparent definitions of all planned outcome measures (Dickersin, 1992; Simes, 1986). All studies that prospectively assign human participants to one or more interventions should be registered, regardless of phase, setting, intervention, and outcome (World Medical Association, 2001). Trial registration is a long-standing practice in clinical medicine, with the International Committee of Medical Journal Editors requiring prospective trial registration as a condition for publication since 2005 (De Angelis et al., 2004; De Angelis et al., 2005). Because trials funded by the NIH after 2019 must be registered and their results must be reported on ClinicalTrials.gov, the practice of registration is expected to increase in prevention science and related disciplines. Prevention scientists conducting trials with health outcomes can use the ClinicalTrials.gov (Zarin et al., 2016), while those working on non-health topics may prefer subject-specific registries such as the Registry of Efficacy and Effectiveness Studies in education (Spybrook et al., 2019) or the American Economic Association registry.

As recommended in Standard 8 of the SPR Standards of Evidence for Efficacy (Gottfredson et al., 2015), prevention scientists can consult reporting guidelines when writing manuscripts to ensure accurate representations of their intervention evaluations (Morris & Clark, 2013). For example, the Consolidated Standards for Reporting Trials (CONSORT) Guidelines have been officially endorsed by over 600 journals that implement these guidelines as part of manuscript submission, peer-review, and editorial decision-making (Shamseer et al., 2016). The CONSORT extension for Social and Psychological Interventions (CONSORT-SPI) identifies the minimum information needed to understand and apply the results of randomized controlled trials (RCTs) that evaluate interventions thought to work through social and psychological mechanisms of action (Montgomery et al., 2018). To facilitate adherence, the user’s manual provides guidance tailored to concepts, theories, and taxonomies used in the social and behavioral sciences (Grant et al., 2018).

### Translational Research, Policy, and Practice

Translational research is the intersection between prevention science and public policy, in which insights from epidemiological and interventional research inform real-world policies and practices that promote individual and collective well-being. Public policy research includes not only researchers within academic institutions, but also individuals located within government agencies, nonprofits, and other settings where research is often inaccessible due to journal paywalls. Open information systems are critical in building and using knowledge management systems to advance dissemination and implementation science (Chorpita & Daleiden, 2014). Preprints and open access articles allow consumers of evidence and practitioners to have more direct access to findings and (in the case of preprints) make evidence more timely. Decisions to scale particular evidence-based programs often are based on windows of opportunity and funding allocations, introducing higher-stakes in the decisions about using evidence (Fagan et al., 2019). This higher-stake nature of using evidence makes open science practices even more important (Supplee & Meyer, 2015). Registration can support testing the reproducibility of research on innovations, as it includes the details as a necessary first step in those processes. Prespecified tests would earn more confidence from the public policy community—and therefore, more utility in decisions around scaling-up programs. Study registration is also critical for high-quality research synthesis that informs policy. Currently, conclusions drawn from meta-analysis and systematic reviews can be limited by “closed” primary research, as reviews cannot fully assess the extent to which particular programs have been tested and not found to be significant. Finally, reproducible workflows in combination with archiving data and code could allow the necessary reproducibility to increase confidence in findings and whether to scale a particular program.

### Innovative Methods and Statistics

Open science can advance prominent methods and approaches in prevention science, such as community-based participatory research, qualitative methods, and administrative data.

#### Community-Based Participatory Research

Community-based participatory research (CBPR) entails unique challenges and opportunities for transparency and openness, given shared power structures with non-scientists, use of a broad range of methods, concerns about privacy, and unstructured data. The forward planning and transparency demands of the open science movement may initially seem like an anathema to prevention scientists working in the context of partnerships with communities in the development, evaluation, and dissemination and scale up of preventive interventions; however, open science practices could improve capacity for ongoing communication, transparency, accountability, reliability, and reciprocity in relationships with community stakeholders across all phases of prevention science. Discussion of ethics in community-based or participatory-action research have identified the clear need for openness and transparency and for ongoing review of assumptions and objectives throughout the lifecycle of a research-practice partnership (Hiriscau et al., 2014; Leadbeater et al., 2006; Leadbeater et al., 2018; Solomon et al., 2016; Tamariz et al., 2015). Rather than constraining action, open science approaches may offer a structure for establishing agreements about key expectations, workflow, data-sharing, dissemination, and reproducing findings, and for reviewing and revising these agreements as the research progresses. For example, collaborative partnerships between researchers and community members can include equitable access to and use of datasets (Gennetian et al., 2020).

Several challenges of the CBPR process could be anticipated and avoided by following open science principles that could lead the partnership systematically, through a series of planning discussions that are open and transparent, not only to the researchers, but also to their community partners. To date, the open science movement has focused primarily on the need for transparency in relation to statistical problems of defining research hypotheses, data collection, workflow for analyses, data sharing, and reproducibility. However, jointly clarifying research and community goals for a project at the outset could also enhance overall project transparency and reproducibility. For example, written agreements could be beneficial in bridging academic and community cultural gaps by jointly considering:

1. registration of agreed plans to clarify aspirations, objectives, and expected outcomes;
2. specifying how work will progress and what timelines are realistic;
3. delineating plans for data analysis, ownership, sharing, and publication;
4. reviewing cultural values and ethical concerns that guide the partnership and define limits of the partnership and protections for vulnerable individuals and communities;
5. defining the scope of independent and collaborative roles in adapting, controlling, and implementing knowledge gained from the partnership; and
6. creating mechanisms for reproducibility (e.g., manuals, protocols, codebooks) so that communities not originally involved can benefit from the knowledge generated.

Funding to do this up-front work is also more likely if it is clearly spelled out in a systematic framework and connected to defining the nature of the community-based collaboration. While an open science approach may not be the only way to organize this foundational knowledge for community-based research, following the intent of open science to improve the transparency and clarity of research partnerships with community partners may strengthen these relationships and the quality of the research produced through their collaborations.

#### Qualitative Research

Given the amount of attention to experimental and quantitative approaches, open science practices present unique epistemological and methodological issues for qualitative and mixed-methods research (Chauvette et al., 2019; Pownall et al., 2021). Qualitative scholars are exploring how open science from hypothetico-deductive frameworks can be translated to qualitative inquiry and its commitments to validity, transparency, ethics, reflexivity, and collaboration (Humphreys et al., 2021). For example, rather than being used to establish experimental predictions, registration could define the aims of a project, outline presuppositions, be updated as data are collected and analyzed to track the development of the interpretative framework, and combat dissemination biases in the qualitative literature (Haven & Van Grootel, 2019; Lorenz & Holland, 2020). Qualitative researchers can aspire to share materials like detailed memos, codebooks, and information on inter-rater reliability (Lorenz & Holland, 2020). Qualitative researchers are also demonstrating ways in which data management plans can be developed to share various forms of data—such as photos, audio recordings, interview transcripts, and field notes—in an ethically and legally appropriate manner (Antonio et al., 2020). Prevention scientists could contribute empirical examples to the nascent but dynamic literature on making qualitative research more transparent and open (Kapiszewski & Karcher, 2021).

#### Administrative Data

Administrative data involve information that organizations routinely collect to monitor and evaluate how well their operations achieve their intended goals (Goerge et al., 2017). For example, McNeely et al. (2019) created a panel dataset of all students enrolled in a public school in a metropolitan county in a Midwestern state between 2004 and 2015 by linking data from the state’s Department of Education, the state’s Department of Human Services, and the county attorney’s office. With this dataset, they conducted a quasi-experimental difference-in-differences analyses to evaluate long-term effects of a truancy diversion program on school attendance. The decreasing costs of obtaining big datasets, combined with improved technology, make research using administrative data easier to conduct over time. While these advances allow for higher-powered analyses, they also risk spurious findings if multiple results are calculated but reported incompletely (Huffman, 2018). Prevention scientists using administrative data would gain efficiency and accuracy in their research processes by leveraging principles of data and computational science with powerful, existing open source software. The study of computational reproducibility is an emerging area, powered by recent advancements in computational and data sciences (Stodden et al., 2016). Although other social and behavioral disciplines have made advancements in these areas, these computational principles and tools have yet to gain a strong foothold in prevention science. For example, research using administrative data would benefit from organized workflows with consistent and predictable structures. A basic research study with a reproducible workflow would contain a folder structure for storing analytic code, raw data, processed data, outputs, and narrative reports using version control (Wilson et al., 2017). Project folders contain a “README” file” that describes each folder in sufficient detail for another researcher to understand their contents and how to reproduce any analyses generating processed data, outputs, and narrative reports. The DRESS (Documenting Research in the Empirical Social Sciences) Protocol provides a set of standards for organizing and documenting workflows for reproducibility purposes (Project TIER, 2016). Projects in RStudio, with the R programming language, provide an excellent starting point to build reproducible workflows for each prevention research project or manuscript and are easily extensible to other collaborative and interactive programmatic tools such as web applications (Gandrud, 2013; Kitzes, 2018).

## Leveraging Prevention Science to Advance the Open Science Movement

Open science proponents often refer to their work as “meta-science” or “meta-research,” i.e., the scientific study of science itself in order to evaluate and improve research practices (Ioannidis et al., 2015; Munafò et al., 2017). Following a translational framework of science (Hardwicke et al., 2020), open science reforms require a broad communal effort, involving a collaborative ecosystem of scientists, research institutions, journals, funders, and other stakeholders across disciplines and countries to change researcher behaviors and scientific culture (Holzwart & Wagner, 2020). A “one-size-fits-all” approach therefore will not be effective: multiple measures must be identified, tailored, and implemented from both the “top-down” and the “bottom-up” (Academy of Medical Sciences, 2015).

Prevention science is well-positioned to engage with the open science movement, given its focus on examining and addressing complex social and behavioral issues. Prevention scientists have unique expertise in socio-ecological, systems-based, context-sensitive approaches needed to identify, develop, and implement open science reforms (Fawcett et al., 2000). For example, open science efforts can be operationalized and approached using established frameworks for intervention development, evaluation, and implementation (Craig et al., 2008). In terms of intervention development, the design, conduct, analysis, and reporting of any study can be seen as behaviors of researchers embedded within a complex social system of stakeholders (Norris & O’Connor, 2019). Open science efforts, therefore, are an attempt to change behavioral and social causes of problems in the research process, requiring the use of tools from behavior change interventions and complex social systems science to help stakeholders adopt desired practices across the research lifecycle (Bartholomew Eldredge et al., 2016; Michie et al., 2014). Once designed, theories from implementation science can be used to identify potential facilitators and obstacles to the delivery of open science efforts (Atkins et al., 2017). Once implemented, interventions to promote open science should be evaluated rigorously to examine whether they are delivered as intended, achieve desired effects, and avoid unintended negative consequences (Craig et al., 2017; Moore et al., 2015).

Compared to other applied disciplines, prevention scientists could be particularly helpful to the open science movement through the use of program planning models to rigorously develop, organize, and guide strategic actions intended to improve transparency, openness, and reproducibility (Green & Kreuter, 2005). That is, the motivations for and efforts of the open science movement can be conceptualized as problem and program theory, with a continuum of interventions to promote open science across primary, secondary, and tertiary levels of prevention (see Fig. 2). Adapting a disease prevention perspective, the distal outcome of the open science movement can be conceptualized as the prevalence of reported research findings that are false (Ioannidis, 2005). A key issue perceived to have a significant impact on or contribute significantly to this distal outcome is the irreproducibility of research findings. The behavioral and social determinants of this issue are selective non-reporting, research misconduct, and misaligned incentives in the scientific ecosystem (Ioannidis, 2014). A key factor enabling these behavioral and social determinants is the traditionally “closed” lifecycle of human subjects research in the health, social, and behavioral sciences. Following this problem theory, open science efforts can be framed positively using the “program theory” of a strengths-based intervention approach (Staudt et al., 2001). That is, rather than assuming malicious intent and policing bad behavior, the ultimate goal of the open science movement can be conceptualized from a health promotion perspective as protecting and further advancing the value of (Macleod et al., 2014) and public trust in science (National Academies of Sciences, 2017). Key distal outcomes include increasing the prevalence of research findings that are “true” as the indicator for more rigorous and reliable bodies of research (Ioannidis, 2014), as well as promoting more inclusive creation of scientific knowledge and accelerated scientific progress (National Academies of Sciences, 2018). This program planning model can underpin an iterative, continuous quality improvement process that ensures open science efforts are theoretically sound, empirically based, and outcome-oriented.

## Potential Challenges of a Transparent, Open, and Reproducible Prevention Science

Challenges to the movement toward a transparent, open, and reproducible prevention science include both warranted concerns and misconceptions (see Table 3). For example, prevention science commonly involves collecting sensitive personal information from vulnerable populations. This requires special care to ensure that sharing de-identified data, code, and materials does not increase risks to participants through violations of privacy and confidentiality (Grant & Bouskill, 2019). In addition, researchers have expressed concern about work being “scooped,” excessive criticism by others, and tension with intellectual property restrictions in the context of transparent, open research (Gilmore et al., 2020). To allay these concerns, appropriate embargo periods could provide researchers with protected time to be the first to analyze their data and publish findings, followed by appropriate rewards for sharing and citation of data, code, and materials after this embargo period (Gennetian et al., 2020; Moher et al., 2018). Open science reforms also need to avoid reinforcing existing inequitable power structures by ensuring stakeholders from under-resourced settings (Nabyonga-Orem et al., 2020), historically underrepresented and excluded groups (Dutta et al., 2021; Fox et al., 2021; Sabik et al., 2021), and diverse epistemic backgrounds (Devezer et al., 2019; Siegel et al., 2021) are included in influential reform discussions. Moreover, proponents need to address concerns about the potential for open science to add burdensome bureaucracy and regulation, stifle creativity and discovery, and be wholly inappropriate outside of the hypothetico-deductive model (Academy of Medical Sciences, 2015). Lastly, proponents need to attend to the potential for open science practices to falsely signal quality and result in the same problems they aim to address (Gorman et al., 2019). Concerted, meaningful discussion about these reservations are needed to yield sustained uptake of open science practices among prevention scientists.

**Table 3:** Potential Reservations about Open Prevention Science: A Tool for Promoting Discussion

**Reservation**	**Response**
**Open Science Generally**	
The field of prevention science is so different from clinical medicine and lab-based experiments that “open science” doesn’t really fit with the type of work we do	Open science is for all fields of science. The goal of open science is to make the scientific process (rationale, design, methods, statistical approaches) transparent and the results more accessible to scientific and public audiences. This goal is especially important for prevention science to fulfill its mission because it must be accessible and trusted by policymakers and the public
Open science dictates one type of scientific study for everyone and restricts academic freedom and discovery	Open science is a set of principles supporting transparency in scientific discovery across scientific methods. These principles can be operationalized in ways sensitive to the underpinnings of each type of study and that respect exploratory work
**Study Registration**	
Prospective registration doesn’t work for prevention science because it is difficult to predict all the possible outcomes that might result for a preventive intervention (particularly over the life course), and it precludes exploratory work (like subgroup effects)	Prospective registration does not preclude the addition of outcomes over the course of a study. Rather, it transparently documents which research questions, hypotheses, outcomes, and analyses were planned at which points in time of a research project
Study registration isn’t appropriate or needed for descriptive or epidemiologic studies; it is really only relevant for research using hypothesis testing approaches such as RCTs	Study registration can be useful to document the existence and link products for any empirical study. While prospective registration of study protocols and analysis plans is an established practice in randomized trials, researchers using other study designs are discovering benefits to the transparent documentation of the planned research approach prior to study initiation
**Data Sharing and Archiving**	
Data archiving is an expensive and burdensome process, particularly because it typically happens after the award ends when the grant has already closed out and there is no funding left to support it	While it is true data archiving has costs, many funders are beginning to either require or encourage the practice, opening the potential for archiving processes to be built into grant budgets and timelines
I don’t have the time or staffing to respond to questions or requests for data files from old projects, and it is too much work to do all of this extra stuff to make my files available to other researchers	Archiving data following best-practices can improve the quality of the data available to external parties, minimizing the amount and intensity of requests. Planning for data archiving at the outset of projects can minimize the “extra” amount of work involved
I am concerned that if I archive my data sets, someone will try to scoop me before I have had a chance to publish my main findings or supplemental studies	Several platforms allow for archiving data with an embargo period, providing researchers with protected time to publish their findings after self-archiving
I am concerned that, if I make my data files and code available to others, then someone may try to prove me wrong, make me look bad, or imply I am unethical or biased in my reporting of prior findings	As with any principles, open science practices have the potential for competitive use or personal gains at the expense of others. The documentation of research rationales, designs, methods, data, and analyses may afford protection against nefarious charges
I worked hard to collect all these data, and my collaborators and study partners trust me to keep the data private	There are sophisticated methods for protecting the privacy of data (e.g., data masking, pseudonymization, data generalization, and synthetic data creation)
These data reflect years of my effort and energy. Why would I want to just turn them over to anyone else?	Advances in science rely on shared information across researchers and disciplines. Rather than just publishing findings, open science advocates for sharing additional scientific products like data to expand discovery
**Privacy and Ethics**	
Our consent forms did not include the archiving of data for future research use, so we cannot archive or share data	Research conducted in the past needs to follow the data sharing permissions granted in the informed consent. Going forward, investigators should use informed consent templates that allow for future research use
My IRB or study participants won’t let me archive or share my data, as it is too sensitive	Open science principles encourage data sharing but not at the expense of privacy and confidentiality. There may be some data that is sensitive or subject to privacy concerns. However, much of the data collected in prevention science can be shared in fully de-identified form, while protecting privacy and confidentiality
I work with Indigenous groups who own data collected and do not want it shared	Research conducted in partnership with Indigenous groups should discuss data sharing as part of study preparation activities. Teams should respect Indigenous Peoples’ rights to control, access, and govern their data
Genetic data cannot be used for research outside of its intended purpose and therefore cannot be shared, so open science rules cannot apply to these data	Ethical principles in the use and sharing of genetic data are unique and should be clearly discussed in study planning and transparent in consent forms
**Impact on the Future of Prevention Science**	
Prevention studies are not sufficiently funded or resourced to do these additional types of open science activities	As support for open science grows, public and private funders are increasingly including allowances or requirements for open science practices and providing grant support for these activities. In addition, many open science practices are already part of standard research practices, such as clarifying study designs, publishing study protocols, and pre-analysis plans in grant applications
The effect sizes for many preventive programs are often small, reflecting the complexity of our work. As such, the application of open science standards has a strong potential to undermine our findings and funding for future prevention research	Prevention science is dedicated to a rigorous process for identifying evidence-based practices using high standards of evidence. Where effect sizes are not robust, this might suggest the need to further enhance the impact and potency of preventative interventions to yield larger impacts
Restrictive rules can set the field back, particularly with regard to public perspective on the impact of prevention, much less scale up of any programs previously thought to be “effective.”	Evidence-based policy depends on the trust and understanding that decision-makers and the public have in research. Support for evidence-based policy will continue if it produces strong replicable outcomes. Open science practices can support these goals
**Incentives**	
My university doesn’t give “credit” for engaging in open science, publishing in open access journals, posting pre-prints, or sharing data. I can’t spend a lot of my time doing something that doesn’t count for promotion	Many open science practices can increase researchers’ impact on the field through broader dissemination of findings, engaging with other researchers in collaboration and dialog, and increasing professional reputations
Early-career scholars will be negatively affected by having to follow all these new requirements	The science of early-career scholars has the potential to be strengthened through increased collaboration, increased public awareness and value for their science, and the use of resources such as open methods and open data to advance their science

## Conclusion

We have identified open science activities that could strengthen the reliability and efficiency of prevention science, facilitate access to its products and outputs, and promote collaborative and inclusive participation in research activities. Overall, we contend that prevention scientists are well-positioned to engage with the open science movement, especially given their expertise in designing solutions for complex social and behavioral problems. In addition, because prevention scientists intervene in the lives of research participants and seek to impact the lives of others, they are scientifically and ethically obligated to conduct and report research in a manner that is likely to produce accessible, true results. Prevention science can better achieve its mission to advance the promotion of individual and collective well-being by identifying ways to engage with principles of transparency, openness, and reproducibility.
